# Pediatric obesity is associated with an altered gut microbiota and discordant shifts in *F* *irmicutes* populations

**DOI:** 10.1111/1462-2920.13463

**Published:** 2016-08-22

**Authors:** Alessandra Riva, Francesca Borgo, Carlotta Lassandro, Elvira Verduci, Giulia Morace, Elisa Borghi, David Berry

**Affiliations:** ^1^ Department of Microbiology and Ecosystem Science, Division of Microbial Ecology Research Network Chemistry Meets Microbiology, University of Vienna Althanstrasse 14 Vienna Austria; ^2^ Department of Health Sciences Università degli Studi di Milano via di Rudinì, 8 Milan Italy; ^3^ Department of Pediatrics San Paolo Hospital via di Rudinì, 8 Milan Italy

## Abstract

An altered gut microbiota has been linked to obesity in adulthood, although little is known about childhood obesity. The aim of this study was to characterize the composition of the gut microbiota in obese (*n* = 42) and normal‐weight (*n* = 36) children aged 6 to 16. Using 16S rRNA gene‐targeted sequencing, we evaluated taxa with differential abundance according to age‐ and sex‐normalized body mass index (BMI *z*‐score). Obesity was associated with an altered gut microbiota characterized by elevated levels of *Firmicutes* and depleted levels of *Bacteroidetes*. Correlation network analysis revealed that the gut microbiota of obese children also had increased correlation density and clustering of operational taxonomic units (OTUs). Members of the *Bacteroidetes* were generally better predictors of BMI *z*‐score and obesity than *Firmicutes*, which was likely due to discordant responses of *Firmicutes* OTUs. In accordance with these observations, the main metabolites produced by gut bacteria, short chain fatty acids (SCFAs), were higher in obese children, suggesting elevated substrate utilisation. Multiple taxa were correlated with SCFA levels, reinforcing the tight link between the microbiota, SCFAs and obesity. Our results suggest that gut microbiota dysbiosis and elevated fermentation activity may be involved in the etiology of childhood obesity.

## Introduction

The gut microbiota is involved in the regulation of multiple host pathways and participates in metabolic and immune‐inflammatory axes connecting the gut with the liver, muscle and brain. The gut microbiota co‐develops with its host from birth and is subjected to a complex interplay that is influenced by host genome, nutrition and lifestyle (Nicholson *et al*., 2012). Diet can have a particularly marked impact on the gut environment, affecting factors such as gut transit time and pH. In particular, alterations in the intake of carbohydrates, proteins and fats can significantly affect the composition of the microbiota (Scott *et al*., 2013). One of the main activities of the gut microbiota is to break down substrates such as resistant starch and dietary fiber, which are incompletely hydrolysed by host enzymes in the small intestine. The main fermentation products resulting from fiber breakdown are the short chain fatty acids (SCFAs) acetate, propionate and butyrate, which play different roles in energy salvage (Schwiertz *et al*., 2009). Microbially‐derived SCFAs provide an additional source of energy for the body: propionate is taken up by the liver and used as a precursor for liponeogenesis, gluconeogenesis and protein synthesis; acetate is used as a substrate for cholesterol synthesis; and butyrate is the main energy supply for colonic epithelial cells (Kallus *et al*., 2012).

The adult human gastrointestinal tract microbiota has been extensively studied in relation to its role in gut homeostasis and various diseases (Schwiertz *et al*., 2009). Notably, alterations in the gut microbiome and metabolome have been associated with the development of obesity (Choquet *et al*., 2010; Vinolo *et al*., 2011). Obesity is a multifactorial disease that predisposes to several comorbidities (Ang *et al*., 2013) and is considered to be a global epidemic by the World Health Organisation (Schwiertz *et al*., 2009). In recent years, the prevalence of childhood obesity has increased substantially worldwide, and currently 23% of children and adolescents in developed countries can be classified as overweight or obese (Ng *et al*., 2014).

Information regarding the structure and function of the gut microbiota during childhood is limited. Although it has been suggested that the microbiota reaches a relatively stable adult‐like state in the first three years of life, other evidence indicates that it continues to develop through adolescence (Hollister *et al*., 2015). As such, childhood may provide unique opportunities for microbiota interventions to promote health or prevent disease. It is, therefore, vital to establish a baseline understanding of pediatric gut microbiota structure and function, as during this period the gastrointestinal tract undergoes a transition from an immature to a mature state (Hollister *et al*., 2015).

The goal of the present study was to characterize the composition of the gut microbiota in obese and normal‐weight children using 16S rRNA gene‐targeted sequencing. We recruited a large cohort of children from the same geographic area to reduce variation unrelated to obesity. We compared gut microbiota profiles with SCFAs and BMI *z*‐scores to gain insights into the structure and activity of the microbiota in pediatric obesity.

## Results

### Pediatric cohort characteristics

A total of 78 children were enrolled at the Pediatric Department of San Paolo Hospital, Milan, Italy. Fecal samples were collected from 36 normal‐weight (N) and 42 obese (O) children (N, BMI *z*‐score: −2.12 to 1.56; O, BMI *z*‐score: 2.14–5; *p* < 0.0001). Cohort characteristics, including age, sex, BMI *z*‐score, mode of delivery in childbirth and history of breastfeeding or formula feeding as an infant were considered (Supporting Information Table S1). There was no significant relationship between history of breastfeeding or formula feeding as an infant with obese and normal‐weight classification (Chi‐square test; *p* = 0.610). Children born by Caesarean section tended to be obese, although this trend did not reach statistical significance at the *p* = 0.05 level (Chi‐square test; *p* = 0.068). Dietary habits were also collected and obese children showed higher dietary intakes of energy and macronutrients (proteins, carbohydrates, sugars and fats) compared to normal‐weight subjects (Supporting Information Table S3).

### SCFAs are increased in the stool of obese children

We observed significantly higher concentrations of acetate, propionate and butyrate, as well as total SCFAs, in the stool of obese compared to normal‐weight subjects (*p* < 0.05 for all comparisons; Supporting Information Table S2). Moreover, we found that the concentration of total SCFAs was significantly associated with obesity (*p* = 0.0317) and was positively correlated with BMI *z*‐score (*p* = 0.001).

### The intestinal microbiota is altered in obese children

**Figure 1:**
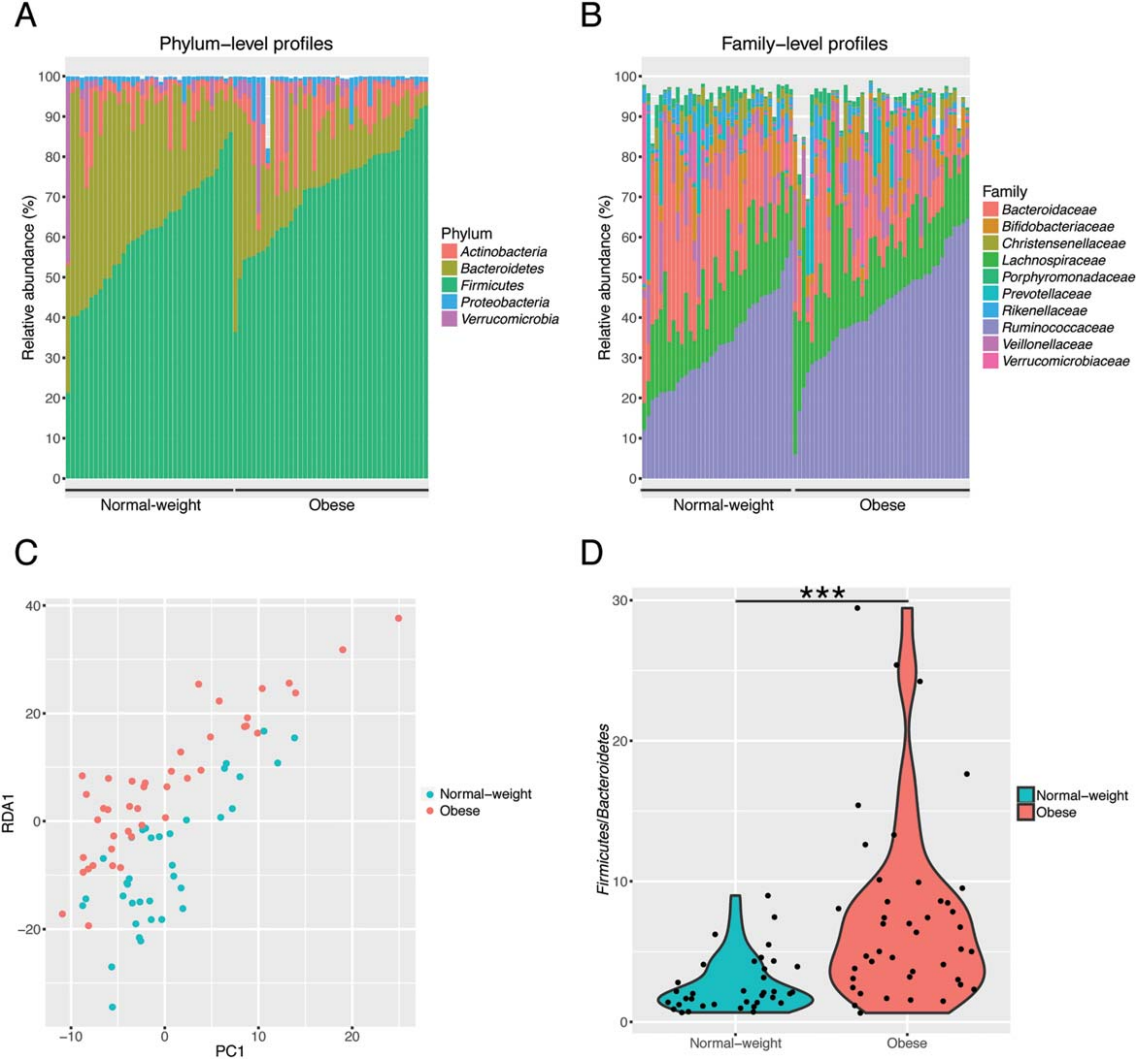
Abundant bacterial taxa in stool samples of normal‐weight (*n* = 36) and obese (*n* = 42) children. Phylum‐level (A) and family‐level (B) taxon profiles are shown. Abundant taxa, defined as having a mean relative abundance of >1%, are shown. C. Redundancy analysis ordination of the gut microbiota according to normal‐weight (blue) and obese (red) groups. D. *Firmicutes*/*Bacteroidetes* ratio for normal‐weight (N) and obese (O) children. The ratio is significantly higher in obese compared to normal‐weight children (*p* < 0.0001).

At the phylum level, the predominant bacterial taxa in feces of both obese and normal‐weight subjects were *Bacteroidetes* and *Firmicutes*, followed by *Actinobacteria*, *Verrucomicrobia* and *Proteobacteria* (Fig. 1A, Supporting Information Table S4). The most abundant families were *Ruminococcaceae*, *Lachnospiraceae*, *Bacteroidaceae*, *Veillonellaceae*, *Bifidobacteriaceae*, *Prevotellaceae*, *Verrucomicrobiaceae*, *Rikenellaceae* and *Christensenellaceae* (Fig. 1B, Supporting Information Table S4). The most abundant genera were *Bacteroides*, *Subdoligranulum*, *Faecalibacterium*, *Dialister*, *Bifidobacterium*, *Pseudobutyrivibrio* and *Blautia* (Supporting Information Fig. S1, Supporting Information Table S4).

The overall composition of the intestinal microbiota, considered at OTU level as well as taxonomic levels ranging from genus to phylum, was significantly affected by obesity, as determined by non‐parametric multivariate analysis of variance testing (perMANOVA; *p* < 0.05 for all levels). Ordination showed that samples from normal‐weight and obese children were distinctly grouped (Fig. 1C). This grouping was confirmed for every taxonomic level by the analysis of similarity (ANOSIM) test, which evaluates significance of sample grouping (*p* < 0.01 for all levels). The intestinal microbiota of obese children was enriched in *Firmicutes* (N: 60.9 ± 14.1, O: 72.1 ± 12.1; mean ± sd) and depleted in *Bacteroidetes* (N: 30 ± 12.6, O: 16.6 ± 11.8) (Supporting Information Table S5). Accordingly, the *Firmicutes*/*Bacteroidetes* ratio was significantly elevated in obese children (*p* < 0.0001; N: 2.6 ± 1.83, O: 7.7 ± 7.1) (Fig. 1D). In agreement with previous observations, the *Firmicutes*/*Bacteroidetes* ratio for obese children displayed a much larger range than for normal‐weight children, which may be partially attributable to the wide range of BMI *z*‐score in the obese group. Consistent with the shifts observed at phylum level, at the family level *Ruminococcaceae* (N: 33.3 ± 11.5, O: 42.5 ± 12.7) was enriched and *Bacteroidaceae* (N: 21.4 ± 12.2, O: 10 ± 7.1) was depleted. At the genus and OTU levels, we observed significant depletion of *Bacteroides* (N: 21.4 ± 12.2, O: 10.5 ± 7.1) as well as *Bacteroides* OTU 7 (best BLAST hit: *Bacteroides vulgatus* with 100% sequence similarity of 422 bp). There were, however, no significant shifts in members of the *Ruminococcaceae* (Supporting Information Table S5).

Gut microbiota richness estimates were not significantly different between samples from obese and normal‐weight children (Observed species: *p* = 0.59; Chao1 estimated richness: *p* = 0.98). Likewise, alpha diversity metrics, which take into account both community richness and evenness, were not significantly different between groups (Shannon: *p* = 0.065; inverse Simpson *p* = 0.34) (Supporting Information Fig. S2). We also found that mode of delivery and infant feeding were not significantly associated with microbiota composition at any taxonomic level (perMANOVA, *p* > 0.05 for all levels). In order to determine whether the higher proportion of Caesarean deliveries among the obese group could impact the difference in microbiota profiles, we grouped the samples according to delivery mode (vaginal or Caesarean section) and calculated if there was a difference in the abundance of taxonomic groups. No significant differences were found at any taxonomic level. Therefore, we conclude that delivery mode did not significantly influence the composition of the microbiota.

### BMI *z*‐score and SCFAs are associated with intestinal microbiota composition

**Figure 2:**
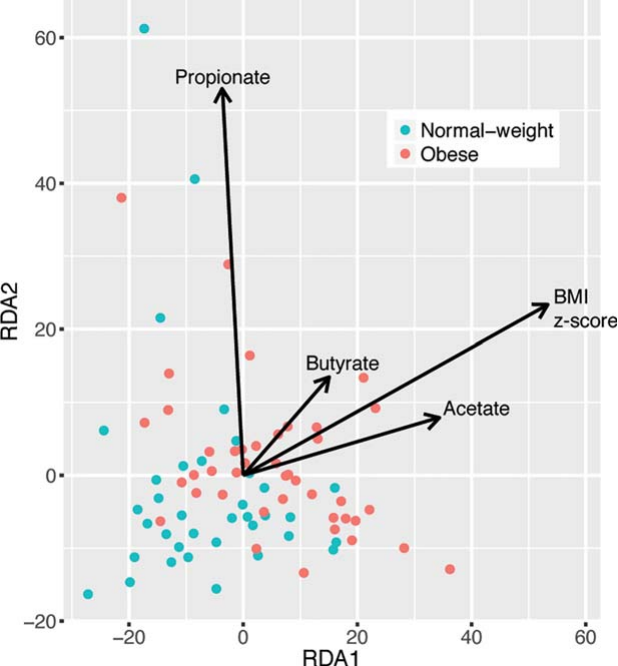
Redundancy analysis of gut microbiota composition with respect to BMI *z*‐score, acetate, propionate and butyrate. The direction of the arrows shows the correlation between variables. Normal‐weight samples are represented by blue dots and obese with red dots.

Childhood obesity is typically defined using age and sex normalized BMI (BMI *z*‐score), to classify subjects into normal‐weight and obese. In order to gain a more fine‐grained understanding of the relationship between the intestinal microbiota and obesity, we evaluated how BMI *z*‐score was associated with microbiota composition. BMI *z*‐score and the SCFAs acetate and propionate were significantly associated with microbiota composition at every taxonomic level (OTU to phylum; *p* < 0.05 at all levels). Additionally, alpha diversity metrics were largely negatively correlated with BMI *z*‐score and SCFA levels (Supporting Information Table S6). We performed redundancy analysis to visualize the relationship between microbiota composition, BMI *z*‐score and SCFAs (Fig. 2). This analysis revealed a strong relationship between BMI *z*‐score and acetate, and to a lesser extent, butyrate levels.

BMI *z*‐score was positively correlated with the abundance of *Firmicutes* (as well as *Ruminococcaceae*) and negatively correlated with *Bacteroidetes* (as well as *Bacteroidaceae* and *Bacteroides*) (Table 1). At the OTU level, *Faecalibacterium* OTU 3 (Best BLAST hit: *Faecalibacterium prausnitzii* with 100% seq. similarity over 402 bp) was positively correlated with BMI *z*‐score, and *Bacteroides* OTUs 7 and 49 (Best BLAST hit: *Bacteroides stercoris* spp. with 99% seq. similarity over 422 bp) were negatively correlated with BMI *z*‐score.

**Table 1:** Bacterial taxa correlated with BMI *z*‐score.

Taxonomic level	Taxon	*R*	*p*‐value
Phylum	*Firmicutes*	0.4145	0.0001
	*Bacteroidetes*	−0.4538	<0.0001
Class	*Clostridia*	0.3688	0.0008
	*Bacteroidia*	−0.4538	<0.0001
Order	*Clostridiales*	0.3687	0.0008
	*Bacteroidales*	−0.4538	<0.0001
Family	*Ruminococcaceae*	0.3778	0.0006
	*Bacteroidaceae*	−0.4930	<0.0001
Genus	*Bacteroides*	−0.4930	<0.0001
OTU	OTU 7: *Bacteroides vulgatus*	−0.4321	<0.0001
	OTU 3: *Faecalibacterium prausnitzii*	0.3058	0.0064
	OTU 49: *Bacteroides stercoris*	−0.3252	0.003

Pearson correlation coefficient (*r*) and *p*‐value are shown for significantly correlating taxa and operational taxonomic units (OTUs).

The abundances of multiple taxa were also correlated with levels of major SCFAs. Several genera within the *Bacteroidetes* were negatively correlated with acetate levels, and multiple genera within the *Firmicutes* were either positively or negatively correlated with acetate (Supporting Information Table S7). At the OTU level, *Faecalibacterium* OTU 3 was positively correlated with acetate. Compared to acetate, the number of correlations was much more limited for propionate and butyrate. The family *Prevotellaceae*, the genus *Prevotella* as well as *Prevotella* OTU 26 (Best BLAST hit: *Prevotella copri* with 99% seq. similarity over 422 bp) were positively correlated with propionate levels. The genus *Faecalibacterium* as well as *Faecalibacterium* OTU 3 were positively correlated with butyrate levels.

### Comparing models to predict BMI *z*‐score based on microbiota composition

We next determined the best microbial predictors of BMI *z*‐score by comparing generalized linear regression models at different taxonomic levels. This revealed that the total explanatory power of the models increased at more refined taxonomic levels (Supporting Information Fig. S3). *Bacteroides* was the main contributor to the genus‐level model (relative importance: 0.172), followed by two genera of the *Ruminococcaceae*, *Faecalibacterium* and *Subdoligranulum* (rel. imp.: 0.08 and 0.03 respectively). At the OTU‐level, the main contributors to the model were *Bacteroides* OTU 7 (rel. imp.: 0.12), *Faecalibacterium* OTU 3 (rel. imp.: 0.08) and *Bacteroides* OTU 49 (rel. imp.: 0.07) (Supporting Information Fig. S3).

### The obese gut microbiota has an altered correlation network structure

**Figure 3:**
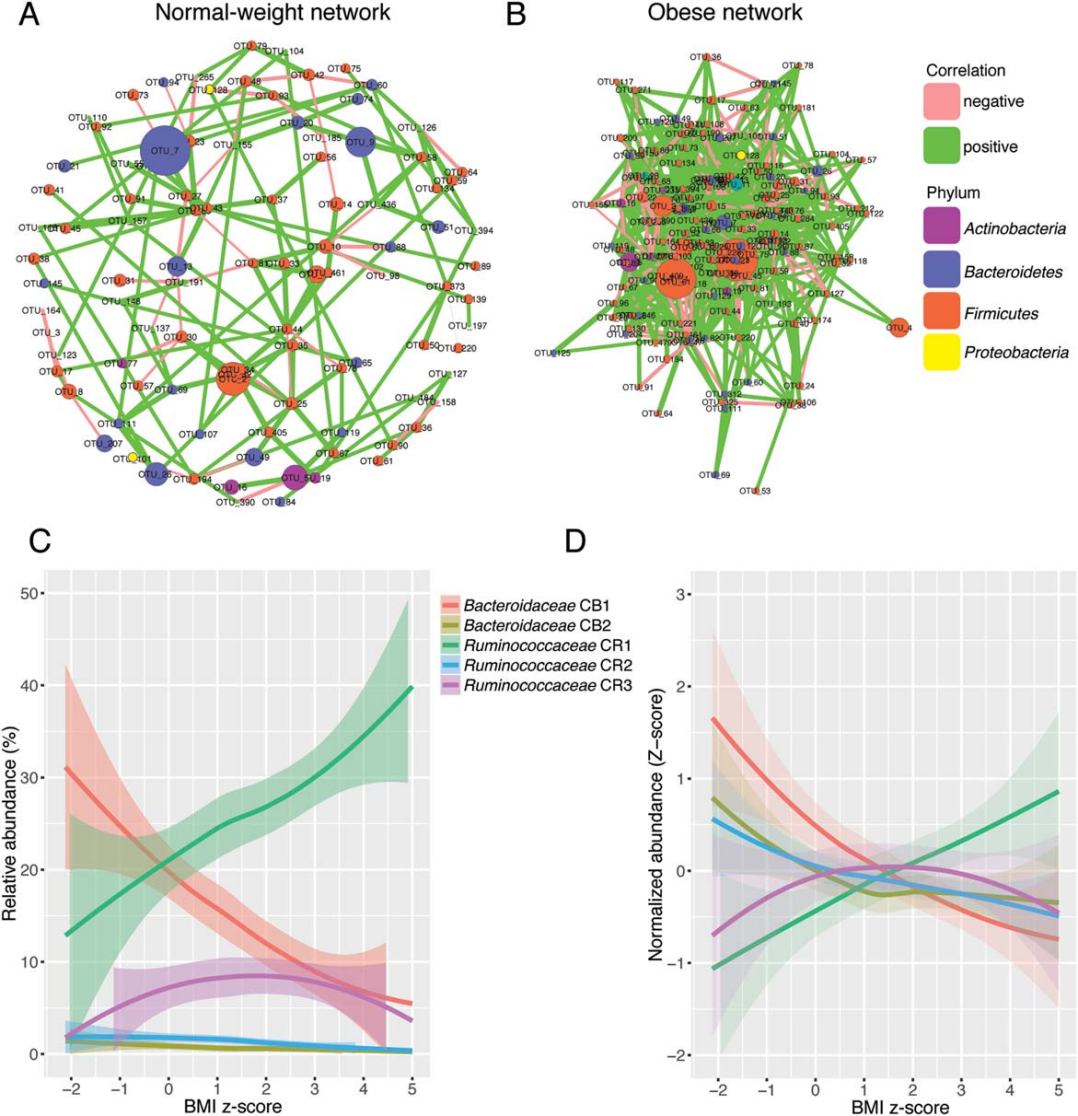
Correlation networks of samples from normal‐weight and obese children. A, B. Networks show significant positive (green) and negative (pink) pairwise correlations between operational taxonomic units (OTUs). OTUs are coloured by phylum affiliation and sized by mean relative abundance. C, D. Correlating communities of *Bacteroidaceae* (CB) and *Ruminococcaceae* (CR) and their abundances with respect to BMI *z*‐score. Relative abundances (C) and *z*‐score transformed abundances (D) are shown. Data points were processed using Lowess smoothing and 95% confidence intervals are shown.

We performed a correlation network analysis to evaluate if obesity was associated with changes in the correlation structure and putative interaction structure of the gut microbiota. We found that networks constructed from samples of normal‐weight children had fewer edges, a lower mean degree and lower transitivity, indicating that there were fewer significant correlations and less clustering of OTUs compared to samples from obese children (Fig. 3A and B; Supporting Information Table S8). The betweenness centrality was higher in normal‐weight sample networks, which indicates that only a few OTUs are highly connected in the network.

We next evaluated whether there were differences in intra‐taxon correlations within the families *Bacteroidaceae* and *Ruminococcaceae*. Interestingly, in both networks *Bacteroidaceae* OTUs with intra‐family correlations were positively correlated with one another, whereas *Ruminococcaceae* OTUs had both positive and negative intra‐family correlations (Supporting Information Table S8). To further explore the difference in intra‐taxon correlations between these groups we extracted clusters of correlating *Bacteroidaceae* and *Ruminococcaceae* OTUs (Supporting Information Table S9). We found that *Bacteroidaceae* OTUs form two communities based on co‐abundance patterns, with the most abundant (*Bacteroidaceae* CB1: 8 OTUs, including OTUs 7 and 49) negatively correlated with BMI *z*‐score and the less abundant not significantly correlated (*Bacteroidaceae* CB2: 2 OTUs). *Ruminococcaceae* was composed of three communities based on co‐abundance patterns, and while the most abundant (*Ruminococcaceae* CR1: 11 OTUs, including OTU 3) was positively correlated with BMI *z*‐score, the second was negatively correlated (*Ruminococcaceae* CR2: 8 OTUs), and the third (CR3: 20 OTUs) was not significantly correlated (Supporting Information Figs. S4 A, B).

## Discussion

The gut microbiota is affected by many factors, such as diet, genetics, health status, environment and lifestyle (Rodríguez *et al*., 2015). Childhood and adult obesity are accompanied by changes in the composition of the gut microbiota (Karlsson *et al*., 2012; Bervoets *et al*., 2013; Borgo *et al*., 2016). In the present study we found alterations in gut microbiota composition and SCFA levels in a cohort of 42 obese and 36 normal‐weight Italian children. We observed that children born by Caesarean section tended to be obese, although this result did not reach statistical significance. Past studies have found that Caesarean section delivery increases the risk of obesity (Goldani *et al*., 2011; Mueller *et al*., 2015; Portela *et al*., 2015) and impacts the infant gut microbiota (Grönlund *et al*., 1999). In our study, delivery mode and infant feeding history (breast‐fed vs. formula‐fed) were not significantly associated with obesity or the gut microbiota composition of children (mean age = 11). The impact of delivery mode and infant feeding history on the gut microbiota may, therefore, be lost after the first years of life, although it is still unclear exactly when (Penders *et al*., 2006; Biasucci *et al*., 2010). Gut microbiota composition has been reported to begin to converge toward an adult‐like microbiota by the end of the first year of life and fully resemble the adult microbiota by 2.5 years of age (Clemente *et al*., 2012), although other studies have shown that the microbiota of children up to 4 years of age differs from that of adults (Kulka *et al*., 2013; Hollister *et al*., 2015), suggesting that conversion to an ‘adult‐like’ microbiota may be a long and gradual process.

Recent scientific advances implicate the gut microbiota as a contributor to over‐nutrition. The gut microbiota enables hydrolysis of indigestible polysaccharides to easily‐absorbable monosaccharides and activation of lipoprotein lipase by direct action of the villous epithelium. Consequently, glucose is rapidly adsorbed and fatty acids are stored in excess (Kalliomäki *et al*., 2008), providing an additional source of energy for the body (Turnbaugh *et al*., 2006). The significantly higher concentration of SCFAs in obese participants in our study may indicate that in obese children colonic fermentation is elevated, or alternatively that there is decreased SCFA absorption due to low‐grade inflammation or more rapid gut transit. This has previously been observed in cohorts of both children (Payne *et al*., 2011) and adults (Schwiertz *et al*., 2009; Fernandes *et al*., 2014). Elevated fecal concentrations of total or individual SCFAs might result from increased microbial production, shifts in microbial cross‐feeding patterns or low mucosal absorption (Schwiertz *et al*., 2009).

We observed a clear alteration in the gut microbiota in obese children at every taxonomic level. This was characterized at the phylum level by an increased abundance of *Firmicutes* and a decreased abundance of *Bacteroidetes* in obese children. It has been hypothesized that an increased ratio of *Firmicutes* to *Bacteroidetes* may contribute to the pathophysiology of obesity and is associated with increased production of SCFAs and energy harvest from colonic fermentation (Turnbaugh *et al*., 2006; Fernandes *et al*., 2014). Although an elevated *Firmicutes*/*Bacteroidetes* ratio in obese subjects has been reported in multiple studies (Turnbaugh *et al*., 2009; Xu *et al*., 2012; Bervoets *et al*., 2013), a reduced *Firmicutes*/*Bacteroidetes* ratio in obese adults has also been found (Schwiertz *et al*., 2009). A recent meta‐analysis concluded that there were no statistically significant differences across multiple studies in the *Firmicutes*/*Bacteroidetes* ratio between obese and normal‐weight adults (Walters *et al*., 2014). In agreement with this meta‐analysis, some pediatric studies have found an increase in *Firmicutes* and a decrease in *Bacteroidetes* (Bervoets *et al*., 2013; Ferrer *et al*., 2013) while others have not (Abdallah Ismail *et al*., 2011; Payne *et al*., 2011). Although in our study the *Firmicutes*/*Bacteroidetes* ratio was significantly elevated in obese individuals, we observed large variation in the ratio, particularly within the obese group. This large variation, as well as the contradicting results from previous studies, suggests that the *Firmicutes*/*Bacteroidetes* ratio may not be a robust marker for obesity.

We reasoned that the classification of individuals into normal‐weight and obese groups might be too coarse of a description for the physiological differences present at different BMI *z*‐scores. We found that the alpha diversity of the gut microbiota was negatively correlated with BMI *z*‐score and we recovered the same broad trends as we observed with obesity classification such as a positive correlation with the *Firmicutes*/*Bacteroidetes* ratio, but with additional insights such as positive correlation of *Faecalibacterium* OTU 3 (*F. prausnitzii*) with BMI *z*‐score and a negative correlation of *Bacteroides* OTU 7 and 49 (*B. vulgatus* and *B. stercoris* respectively) with BMI *z*‐score. *Faecalibacterium*, a group of major butyrate producers in the colon (Louis *et al*., 2009), was also positively correlated with acetate and butyrate, reinforcing the tight link between SCFAs and obesity. Literature data are conflicting about the level of *F. prausnitzii* in obesity, with studies showing positive (Balamarugan *et al*., 2010), negative (Borgo *et al*., 2016) or no association (Feng *et al*., 2014). These contradictory results may be due to experimental factors such as small cohort sizes or the use of different primer sets, or may be explained by the existence of multiple *F. prausnitzii* phylotypes (Louis *et al*., 2009; Hippe *et al*., 2016). Indeed, Hippe and colleagues (2016) suggested that the two identified phylotypes display different physiological properties and seem to produce different amounts of butyrate in the gut. Propionate levels were positively correlated with members of the *Prevotellaceae*, which are known propionate producers (Schwiertz *et al*., 2009), although this was not related to BMI *z*‐score. Interestingly, increase of colonic propionate has been shown to prevent weight gain in overweight adults by stimulating the release of PYY and GLP‐1 from human colonic cells and thereby reducing energy intake (Chambers *et al*., 2014).

In order to determine if the structure of the gut microbiota is also altered in obesity, we performed a correlation network analysis and found that there were fewer correlations and less clustering of OTUs in normal‐weight compared to obese children. The betweenness centrality was higher in the normal‐weight network, which indicates that in the obese microbiota there are more OTUs that are highly connected to other OTUs. It is tempting to speculate that the altered network structure in obese children may be involved in the increased fermentation capacity of the gut microbiota.

Interestingly, intra‐taxon correlations within the families *Bacteroidaceae* and *Ruminococcaceae* demonstrated that in both networks *Bacteroidaceae* OTUs were positively correlated with one another, whereas *Ruminococcaceae* OTUs had both positive and negative intra‐family correlations. This indicates a lack of intra‐family ecological cohesion for *Ruminococcaceae* across these samples and may explain why *Bacteroidetes* taxa were generally better predictors of BMI and obesity than *Firmicutes* taxa. To further investigate the difference between *Bacteroidaceae* and *Ruminococcaceae* responses, we extracted from the complete network the communities of co‐abundant OTUs from these two groups. We identified five distinct correlating communities (2 *Bacteroidaceae* [CB1 and CB2] and 3 *Ruminococcaceae* [CR1‐CR3]). Interestingly, while *Ruminococcaceae* CR1 was positively correlated with BMI *z*‐score, *Ruminococcaceae* CR2 was negatively correlated. The divergent response of members of the *Ruminococcaceae* with respect to BMI *z*‐score may indicate different niche preferences within this group and may also help to explain why the increased *Firmicutes*/*Bacteroidetes* ratio is not found in all studies, as it groups together *Firmicutes* populations with discordant shifts in obesity. Divergent responses of members of the clostridia have previously been observed in other conditions such as inflammation (Berry *et al*., 2012). It is likely that the extensive physiological and metabolic diversity in members of the clostridia is responsible for these contrasting responses, and additional studies are needed to better characterize and functionally categorize the members of this abundant group.

Although it is recognized that the gut microbiota has the potential to change along with the development of its host, information regarding the structure and function of the microbiome in children remains limited (Hollister *et al*., 2015). We hypothesized that an aberrant gut microbiota composition and activity might contribute to the development of childhood obesity. We found that members of the *Bacteroidetes* and certain populations of *Firmicutes* were associated with childhood obesity, although members of the *Firmicutes* exhibited contrasting shifts. Additional studies are needed to better characterize the members of *Firmicutes* and their roles in obesity. Obesity is often associated with altered dietary habits, and in the present study obese children had higher caloric intake. It is therefore not possible to determine if an altered microbiota is a causative factor in pediatric obesity or a consequence of diet, and this must be tested with future research that takes into account diet and physiology and which includes detailed functional analyses of the metabolic activity of the gut microbiota. Together, this will advance our understanding of the role of the gut microbiota in obesity and provide opportunities to improve health and prevent disease.

## Experimental procedures

### Subjects and sample collection

Seventy‐eight children (36 males/42 females, 9–16 years) were enrolled in the study at the Pediatric Department of San Paolo Hospital in Milan from December 2013 to February 2015. The enrollment conditions were performed as previously described (Borgo *et al*., 2016). Briefly, children's BMI was calculated by reported weight/height^2^ (kg m^−2^), and classification of obese (O) and normal weight (N) was made according to Cole (Cole *et al*., 2000). Weight (kg), height (cm) and BMI (kg m^−2^) were transformed to age and sex‐specific *z*‐scores (Cole *et al*., 1995). Inclusion criteria were: children living in Northern Italy born from Caucasian parents with birth weight ≥ 2500 g, gestational age 37–42 weeks and singleton birth. Children with neonatal disease, congenital malformation, antibiotic or probiotic/prebiotic usage in the previous six months, chronic or acute intestinal and obesity‐related co‐morbidity conditions were excluded. Data concerning mode of delivery and type of feeding were collected for all subjects and the dietary habits were assessed at recruitment by means of an age‐adjusted food frequency questionnaire made up of 116 items (Verduci *et al*., 2007). Fecal samples were collected 24 h before medical examination and stored at −20°C until processing.

The study was conducted in accordance with the local medical ethical committee (protocol number 2015/ST/135). Written informed consent was given by a parent for all enrolled subjects.

### DNA extraction and preparation of 16S rRNA gene amplicon libraries

The total bacterial DNA extraction was performed using the Spin stool DNA kit (Stratec Molecular, Berlin, Germany), according to the manufacturer's instructions and amplified by PCR. Amplification was performed with a two‐step barcoding approach according to Herbold and colleagues, 2015. In the first‐step PCR, 16S rRNA genes of all Bacteria were amplified with forward primer S‐D‐bact‐0341‐b‐S‐17 (5‐CCTACGGGNGGCWGCAG‐3′) and reverse primer S‐D‐bact‐0785‐a‐A‐21 (5‐GACTACHVGGGTATCTAATCC‐3′), which also contained head adaptors (5′‐GCTATGCGCGAGCTGC‐3′). In the second‐step PCR, PCR products from the first step were amplified with primers consisting of the 16 bp head sequence and a sample‐specific 8 bp barcode from a previously published list at the 5′ end (Hamady *et al*., 2008). Each PCR reaction (20 μL in first step, 50 μL in second step) consisted of 10× Taq buffer (Fermentas, USA), 2 mM dNTPmix (Fermentas), 25 mM MgCl_2_ (Fermentas), 5 U μl^−1^ Taq DNA polymerase (Fermentas), 20 mg ml^−1^ bovine serum albumin (Fermentas), 50 μM of each of the forward and reverse primers and 5 μl of sample. Thermal cycle conditions were: 95°C for 3 min; 95°C for 30 s, a primer‐specific annealing temperature of 55°C for 30 s, 72°C for 1 min for 25 cycles and an elongation time of 72°C for 7 min (step1); 52°C for 30 s, 72°C for 1 min for 5 cycles (step 2) and an elongation step of 72°C for 7 min. The first PCR reaction was performed in triplicate, pooled for use as a template in the second step and evaluated qualitatively by gel electrophoresis. The barcoded amplicons were purified between the first step and the second step and after the second step with ZR‐96 DNA Clean‐up Kit (Zymo Research, USA) and quantified using the Quant‐iT PicoGreen dsDNA Assay (Invitrogen, USA). An equimolar library was constructed by pooling samples, and the resulting library was sent sequenced on the Illumina MiSeq platform at Microsynth AG (Balgach, Switzerland). Sequence data have been deposited in the NCBI Short Read Archive under SRP073251.

### Short chain fatty acids (SCFAs) measurement

Stool samples were analysed for acetic acid, propionic acid and butyric acid using capillary electrophoresis. For determination of SCFAs concentration one aliquot of frozen fecal sample (50 mg) was used and 200 μl of Milli‐Q filtered water was added. The solution was mixed by vortexing for 10 min and then centrifuged 30 min at 21,000 × g. A standard mix composed of acetic acid, propionic acid, butyric acid, lactic acid, formic acid and succinic acid with consecutive concentration of 50 μM, 100 μM, 250 μm and 350 μM, were run as external standards and calibrated. Caproic acid (100 µM final concentration) was used as internal control. A buffer with 0.01M NaOH, 500 μM CaCl_2_ and 100 μM caproic acid was prepared to run samples. Because we detected interference between phosphates and propionic acid peaks, a final concentration of 500 μM of CaCl_2_ was added in order to precipitate phosphates usually present in human fecal matter. Ceofix Anions 5 kit (Beckman Coulter, USA) was utilized to prepare anion buffers for the machine. SCFAs concentration was determined in 100µl supernatant using P/ACE MDQ Molecular Characterisation System Beckam Coulter (USA) with a fused silica capillary of 75 μm internal diameter × 363 μm outer diameter (Polymicro Tecnologies, USA). Thirty‐two karat software (Beckman Coulter, USA) was used for data processing. SCFAs concentration in fecal samples was expressed in micromoles per gram (µmol g^−1^) of feces.

### Sequence pre‐processing and data analysis

Sequence data were sorted into libraries using the 8 nt sample‐specific barcode and primer using a custom‐made in‐house script, quality‐filtered according to the Earth Microbiome Project guidelines and paired end reads were concatenated (Bokulich *et al*., 2013). Reads were then clustered into species‐level operational taxonomic units (OTUs) of 97% sequence identity, checked for chimeras using USEARCH, and taxonomically classified using the Ribosomal Database Project näive Bayesian classifier (Wang *et al*., 2007). Statistical analysis was performed using the statistical software R (https://www.r-project.org/). To avoid biases related to uneven library depth, sequencing libraries were subsampled to a number of reads smaller than the smallest library (2000 reads). The statistical significance of factors affecting microbiota composition was evaluated using non‐parametric permutational multivariate analysis of variance (perMANOVA), significant clustering of groups was evaluated with analysis of similarities (ANOSIM), ordination was performed using redundancy analysis (RDA) in the vegan package (Oksanen *et al*., 2010). Alpha and beta diversity metrics were also calculated with the vegan package. Indicator species analysis was performed using the indicspecies package (De Caceres *et al*., 2009). Network analysis was performed for all OTUs present in at least 30% of samples as recommended in (Berry and Widder, 2014) using graphical lasso technique cclasso to mitigate biases associated with compositional data (Danaher *et al*., 2014). Network topological and node‐level properties were determined using the igraph package (Csardi, 2015) and networks were visualized using Cytoscape (Shannon *et al*., 2003). Statistical analysis of cohort‐related data was performed using Student's *t*‐test, chi‐square test, correlation analysis (Pearson correlation coefficient) and linear regression modeling. Variables were expressed as mean ± standard deviation (sd), and for multiple comparisons *p*‐values were adjusted with the False Discovery Rate method. A *p*‐value less than or equal to 0.05 was considered statistically significant.

## Conflict of interest

All authors declare no conflict of interest.

## Supporting information

Additional Supporting Information may be found in the online version of this article at the publisher's web‐site.

**Fig. S1.** Abundant bacterial taxa in stool samples of normal‐weight (n=36) and obese (n=42) children. Genus level taxon profiles are shown. Abundant taxa, defined as having a mean relative abundance of >1%, are shown.
**Fig. S2.** Intestinal microbiota richness and diversity in normal‐weight and obese children. Observed species, Chao1 estimated richness, Shannon diversity, and inverse Simpson diversity estimators show no significant difference between the two groups (Observed species: p=0.59; Chao1: p=0.98; Shannon: p=0.065; Inverse Simpson p=0.34).
**Fig. S3.** Generalized linear regression models at different taxonomic levels. (A) The coefficient of determination (R^2^), which indicates the proportion of the variance in the dependent variable that is predictable from the independent variable, increases at genus and OTU levels. (B) The Akaike information criterion (AIC), a measure of the relative quality of statistical models for a given set of data, is lowest at genus and OTU levels.
**Fig. S4.** Correlating communities of *Bacteroidaceae* (CB) and *Ruminococcaceae* (CR) and their abundances with respect to BMI z‐score. Relative abundances (A) and z‐score transformed abundances (B) are shown. Data points were processed using Lowess smoothing and 95% confidence intervals are shown.
**Table S1.** Characteristics of the study cohort. The cohort was composed of normal‐weight (N) and obese (O) children. Body mass index (BMI) was calculated as weight/height^2^ (kg/m^2^), and was transformed to age‐ and sex‐adjusted z‐scores. Values are expressed as mean ± sd. ^a^Information not available for two subjects. ^b^Information not available for three subjects.
**Table S2.** Short chain fatty acid (SCFA) levels in the stool of normal‐weight (N) and obese (O) subjects. Concentrations are calculated as μmol/g wet weight and are expressed as mean ± sd. Total SCFA is calculated as the sum of acetate, propionate, and butyrate concentrations.
**Table S3.** Daily caloric and dietary intake in obese and normal‐weight children. Values are expressed as mean ± sd.
**Table S4.** The relative abundance of abundant bacteria taxa in the study. Abundant taxa are defined as having a mean abundance greater than 1%. *Taxa significantly increased or decreased in obese children (complete details are presented in Table S4).
**Table S5.** Taxa that were increased (+) or decreased (−) in abundance in obese children (O).
**Table S6.** Correlation of alpha diversity metrics with BMI z‐score and SCFAs. Observed OTUs, Chao1 estimated richness, Shannon and inverse Simpson diversity indexes were correlated and the Pearson correlation coefficients (r) and respective p‐values are shown. *indicates p<=0.05 and **indicates p <=0.01.
**Table S7.** Taxa correlated with acetate concentration. The Pearson correlation coefficients (r) and respective p‐values are shown.
**Table S8.** Properties of correlation networks generated from samples from normal‐weight (N) or obese children (O). Nodes are OTUs and edges are significant correlations between OTUs. Other parameters are metrics related to the topology of the network.
**Table S9.** Clusters of correlating *Bacteroidaceae* and *Ruminococcaceae* OTUs extracted from the correlation network. The closest cultured species and its similarity to each OTU (% sequence similarity) are shown.
