# Supplementary material for: Pediatric obesity is associated with an altered gut microbiota and discordant shifts in F irmicutes populations
Source: Environ Microbiol. 2016 Aug 22;19(1):95–105. doi: 10.1111/1462-2920.13463 (PMC5516186; doi:10.1111/1462-2920.13463)
Supplement: Supplementary file 1 — Fig. S1. Abundant bacterial taxa in stool samples of normal‐weight (n=36) and obese (n=42) children. Genus level taxon profiles are shown. Abundant taxa, defined as having a mean relative abundance of >1%, are shown. Fig. S2. Intestinal microbiota richness and diversity in normal‐weight and obese children. Observed species, Chao1 estimated richness, Shannon diversity, and inverse Simpson diversity estimators show no significant difference between the two groups (Observed species: p=0.59; Chao1: p=0.98; Shannon: p=0.065; Inverse Simpson p=0.34). Fig. S3. Generalized linear regression models at different taxonomic levels. (A) The coefficient of determination (R2), which indicates the proportion of the variance in the dependent variable that is predictable from the independent variable, increases at genus and OTU levels. (B) The Akaike information criterion (AIC), a measure of the relative quality of statistical models for a given set of data, is lowest at genus and OTU levels. Fig. S4. Correlating communities of Bacteroidaceae (CB) and Ruminococcaceae (CR) and their abundances with respect to BMI z‐score. Relative abundances (A) and z‐score transformed abundances (B) are shown. Data points were processed using Lowess smoothing and 95% confidence intervals are shown. Table S1. Characteristics of the study cohort. The cohort was composed of normal‐weight (N) and obese (O) children. Body mass index (BMI) was calculated as weight/height2 (kg/m2), and was transformed to age‐ and sex‐adjusted z‐scores. Values are expressed as mean ± sd. aInformation not available for two subjects. bInformation not available for three subjects. Table S2. Short chain fatty acid (SCFA) levels in the stool of normal‐weight (N) and obese (O) subjects. Concentrations are calculated as μmol/g wet weight and are expressed as mean ± sd. Total SCFA is calculated as the sum of acetate, propionate, and butyrate concentrations. Table S3. Daily caloric and dietary intake in obese and normal‐weight childr [file EMI-19-95-s001.pdf]

SUPPLEMENTAL FIGURES AND TABLES

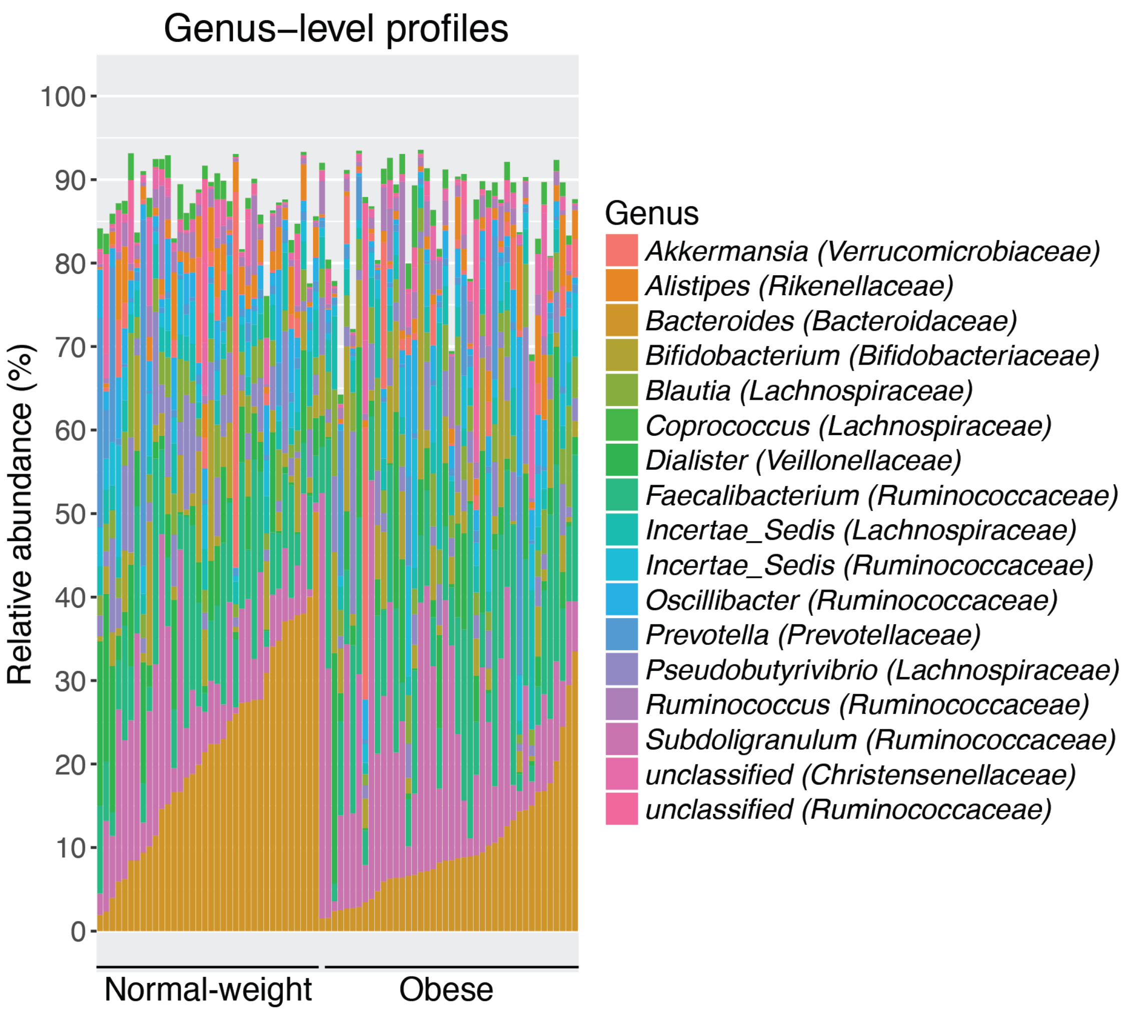

Figure S1. Abundant bacterial taxa in stool samples of normal-weight (n=36) and obese (n=42) children. Genus level taxon profiles are shown. Abundant taxa, defined as having a mean relative abundance of >1%, are shown.

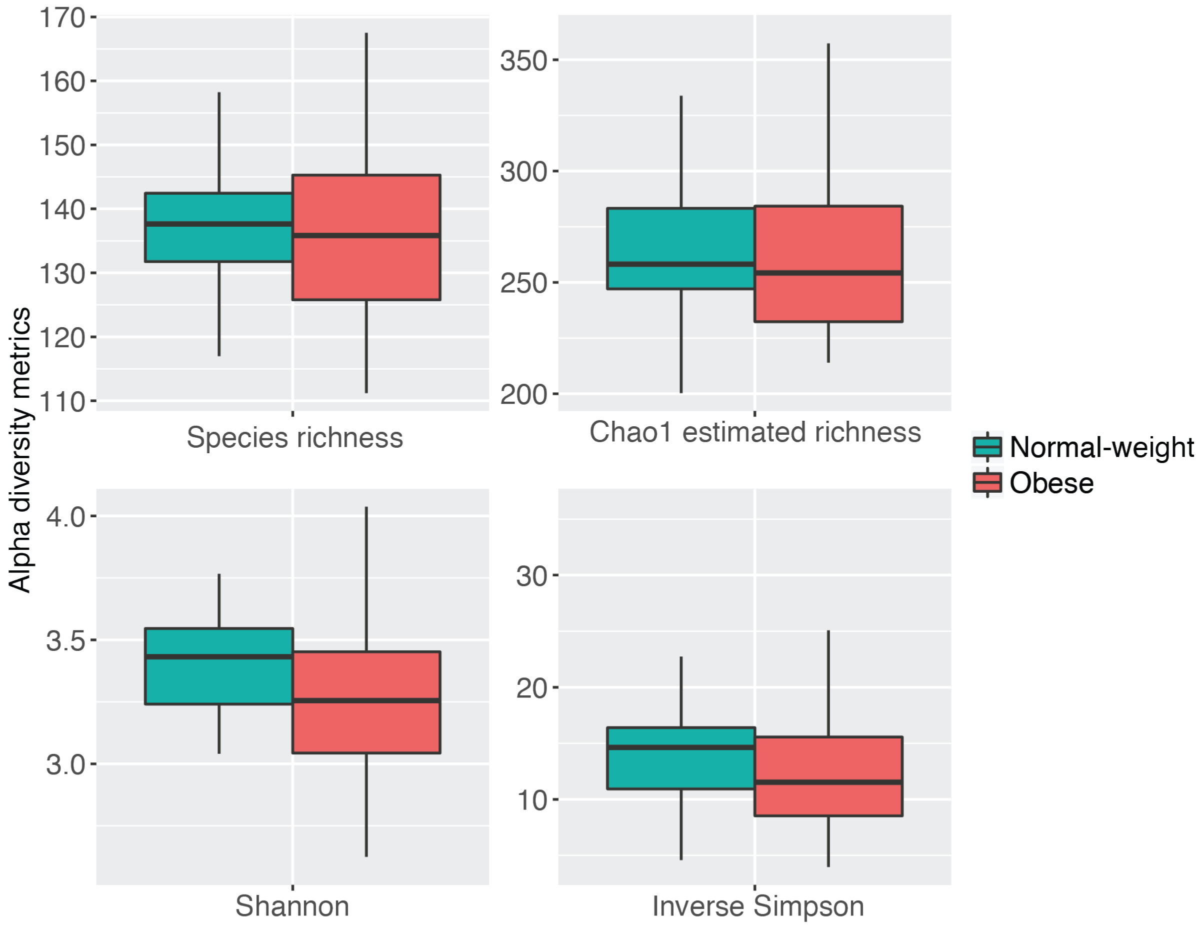

Figure S2. **Intestinal microbiota richness and diversity in normal-weight and obese children.** Observed species, Chao1 estimated richness, Shannon diversity, and inverse Simpson diversity estimators show no significant difference between the two groups (Observed species: p=0.59; Chao1: p=0.98; Shannon: p=0.065; Inverse Simpson p=0.34).

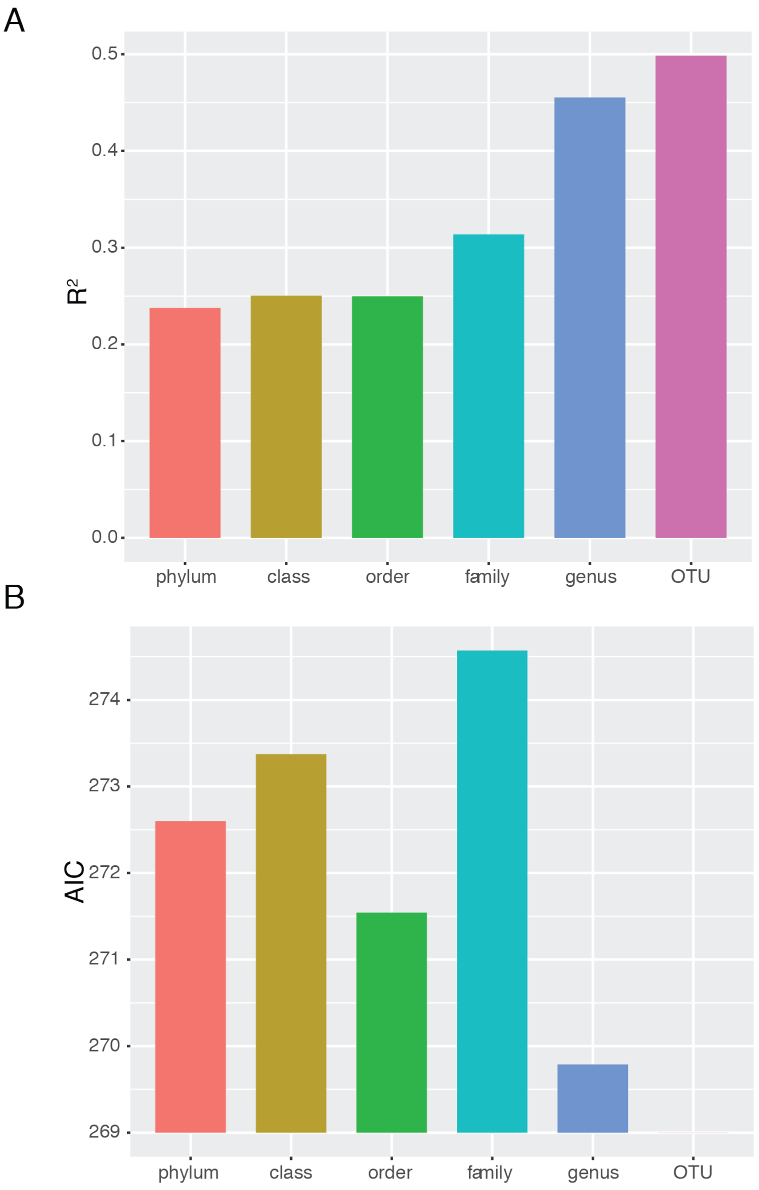

Figure S3. Generalized linear regression models at different taxonomic levels. (A) The coefficient of determination (R^2), which indicates the proportion of the variance in the dependent variable that is predictable from the independent variable, increases at genus and OTU levels. (B) The Akaike information criterion (AIC), a measure of the relative quality of statistical models for a given set of data, is lowest at genus and OTU levels.

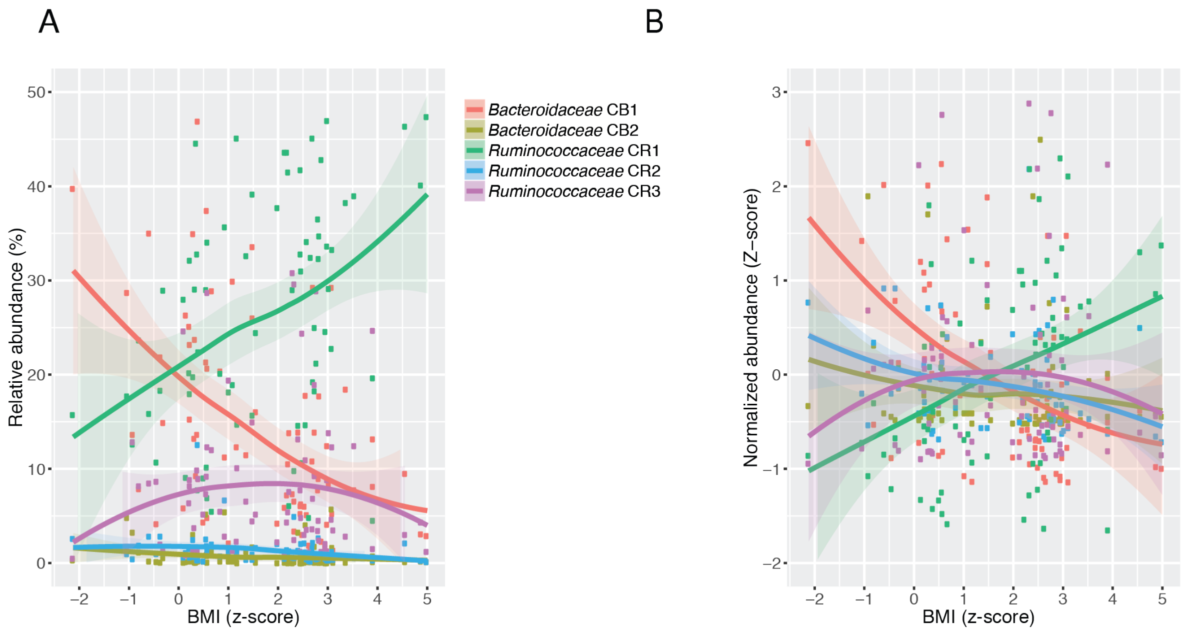

Figure S4: Correlating communities of *Bacteroidaceae* (CB) and *Ruminococcaceae* (CR) and their abundances with respect to BMI z-score. Relative abundances (A) and z-score transformed abundances (B) are shown. Data points were processed using Lowess smoothing and 95% confidence intervals are shown.

Table S1. Characteristics of the study cohort. The cohort was composed of normal-weight (N) and obese (O) children. Body mass index (BMI) was calculated as weight/height^2 (kg/m^2), and was transformed to age- and sex-adjusted z-scores. Values are expressed as mean ± sd. [a]Information not available for two subjects. [b]Information not available for three subjects.

	N (n=36)	O (n=42)
Age (years)	11±0.33	11±1.99
Sex (male:female)	17:19	21:21
BMI z-score	0.3±0.82	3.0±0.7
Delivery type (vaginal: caesarean)	28:8	22:18[a]
Infant diet (breast-fed: formula-fed)	14:22	18:21[b]

Table S2. **Short chain fatty acid (SCFA) levels in the stool of normal-weight (N) and obese (O) subjects.** Concentrations are calculated as μmol/g wet weight and are expressed as mean ± sd. Total SCFA is calculated as the sum of acetate, propionate, and butyrate concentrations.

	N (n=36)	O (n=42)	p-value
Acetate	30.3±13.0	40.4±18.9	0.0081
Propionate	8.8±5.8	12.5±7.7	0.0206
Butyrate	8.4±5.3	12.4±9.8	0.0272
Total SCFA	47.5±20.4	65.3±32.4	0.0049

Table S3. Daily caloric and dietary intake in obese and normal-weight children. Values are expressed as mean ± sd.

Variable	Obese children	Normal-weight children	*p*-value	*p*-value†
Calories (kcal/day)	2264.13 (746.31)	1722.01 (383.78)	0.001 *	0.001*
Protein (g/day)	94.00 (32.76)	68.56 (19.47)	<0.001 *	0.001*
Carbohydrates (g/day)	301.06 (120.66)	201.94 (47.66)	<0.001 *	0.001*
Sugars (g/day)	110.16 (65.70)	75.12 (31.92)	0.005 *	0.012*
Fats (g/day)	82.38 (30.80)	70.02 (18.36)	0.128	0.033*

† Adjusted for age and sex. *Statistically significant

Table S4. The relative abundance of abundant bacteria taxa in the study. Abundant taxa are defined as having a mean abundance greater than 1%. * Taxa significantly increased or decreased in obese children (complete details are presented in Table S4).

		Normal-weight		Obese	
Taxonomic level	Taxon	Mean	sd	Mean	sd
Phylum	*Bacteroidetes**	30	12.6	16.6	11.8
	*Firmicutes**	60.9	14.1	72.1	12.1
	Actinobacteria	5.6	6.1	6.3	4.9
	Verrucomicrobia	2.5	7.6	2.3	5.5
	Proteobacteria	1.2	1.1	2.0	2.9
Family	*Ruminococcaceae**	33.3	11.5	42.5	12.7
	Lachnospiraceae	16.4	6.5	17.9	9.7
	*Bacteroidaceae**	21.4	12.2	10	7.1
	Veillonellaceae	6.1	5.7	6.5	7.5
	Bifidobacteriaceae	5.1	6.0	5.3	4.5
	Prevotellaceae	3.6	8.5	2.8	5.2
	Verrucomicrobiaceae	2.5	7.6	2.3	5.5
	Rikenellaceae	2.8	2.0	2.0	2.6
	Christensenellaceae	2.0	2.3	2.3	3.7
Genus	*Bacteroides**	21.4	12.2	10.5	7.1
	Subdoligranulum	10.7	8.4	16.7	12.3
	Faecalibacterium	10.0	6.3	12.7	8.7
	Dialister	5.9	5.8	5.6	7.1
	Bifidobacterium	5.1	6.0	5.3	4.5
	Pseudobutyrivibrio	5.1	4.1	4.4	3.9
	Blautia	3.6	1.8	4.4	2.4

Table S5. Taxa that were increased (+) or decreased (-) in abundance in obese children (O).

Taxonomic level	Taxon	O	p-value
Phylum	*Firmicutes*	+	0.001
	Bacteroidetes	-	<0.0001
Class	*Clostridia*	+	0.004
	Bacteroidia	-	<0.0001
Order	*Clostridiales*	+	0.003
	Bacteroidales	-	<0.0001
Family	*Ruminococcaceae*	+	0.01
	Bacteroidaceae	-	<0.0001
Genus	*Bacteroides*	-	<0.0001
OTU	OTU 7: *Bacteroides vulgatus*	-	<0.0001

Table S6. **Correlation of alpha diversity metrics with BMI z-score and SCFAs.** Observed OTUs, Chao1 estimated richness, Shannon and inverse Simpson diversity indexes were correlated and the Pearson correlation coefficients (r) and respective p-values are shown. * indicates p<=0.05 and ** indicates p <=0.01.

	Observed OTUs		Chao1 richness		Shannon		Inverse Simpson	
	r	*p*-value	r	*p*-value	r	*p*-value	r	*p*-value
BMI z-score	-0.1562	0.1720	-0.0240	0.8345	-0.2657	0.0186*	-0.1946	0.0877
Acetate	-0.2956	0.0085**	-0.2826	0.0121*	-0.3513	0.0016**	-0.3581	0.0012 **
Propionate	-0.1876	0.1000	-0.2166	0.0567	-0.2584	0.0223*	-0.2575	0.0227*
Butyrate	-0.2387	0.0353*	-0.2701	0.0167*	-0.2639	0.0195*	-0.2354	0.0379*
Total SCFA	-0.2917	0.0095**	-0.3003	0.0075**	-0.3498	0.0016**	-0.3454	0.0019**

Table S7. **Taxa correlated with acetate concentration.** The Pearson correlation coefficients (r) and respective p-values are shown.

Taxonomic level	Taxon	r	p-value
Phylum	*Firmicutes*	0.3107	0.005
	Bacteroidetes	-0.3145	0.005
Class	*Clostridia*	0.2765	0.01
	Bacteroidia	-0.3145	0.005
Order	*Clostridiales*	0.2767	0.01
	Bacteroidales	-0.3145	0.005
Family	*Ruminococcaceae*	0.3120	0.005
	Bacteroidaceae	-0.2876	0.01
	Porphyromonadaceae	-0.2845	0.01
	Rikenellaceae	-0.3107	0.005
Genus	*Ruminococcaceae Incertae sedis (Firmicutes)*	-0.2683	0.01
	Bacteroides (Bacteroidetes)	-0.2876	0.01
	Parabacteroides (Bacteroidetes)	-0.2737	0.01
	Alistipes (Bacteroidetes)	-0.3117	0.005
	Oscillabacter (Firmicutes)	-0.3205	0.004
	Subdoligranulum (Firmicutes)	0.2737	0.01
	Faecalibacterium (Firmicutes)	0.4487	<0.0001
OTU	OTU 3: *Faecalibacterium prausnitzii*	0.4487	<0.0001

Table S8. **Properties of correlation networks generated from samples from normal-weight (N) or obese children (O).** Nodes are OTUs and edges are significant correlations between OTUs. Other parameters are metrics related to the topology of the network.

	**N**	**O**
Nodes	138	143
Edges	370	941
Mean Degree	5.36	13.16
Transitivity	0.047	0.131
Average Path Length	3.10	2.22
Assortativity	-0.0535	0.169
Fragmentation	0	0
Betweeness Centrality	0.0701	0.0226
Closeness Centrality	0.00123	0.00137
Percent Positive corr. in network	69.5	65.5
Bacteroidaceae (intra-family corr.; pos./neg.)	[2/0]	[13/0]
Ruminococcaceae (intra-family corr.; pos./neg.)	[31/5]	[50/18]

Table S9: Clusters of correlating *Bacteroidaceae* and *Ruminococcaceae* OTUs extracted from the correlation network. The closest cultured species and its similarity to each OTU (% sequence similarity) are shown.

Bacteroidaceae			
**Correlating community**	**OTU_ID**	**Closest related species**	**Similarity (%)**
CB1	OTU_7	*Bacteroides vulgatus*	100
	OTU_60	*Bacteroides fragilis*	100
	OTU_13	*Bacteroides caccae*	100
	OTU_51	*Bacteroides thetaiotaomicron*	100
	OTU_49	*Bacteroides stercoris*	99
	OTU_119	*Bacteroides cellulosilyticus*	100
	OTU_9	*Bacteroides uniformis*	100
	OTU_207	*Bacteroides xylanisolvens*	99
CB2	OTU_312	*Bacteroides eggerthii*	99
	OTU_65	*Bacteroides finegoldii*	95
Ruminococcaceae			
**Correlating community**	**OTU_ID**	**Closest related species**	**Similarity (%)**
CR1	OTU_118	*Clostridium leptum*	92
	OTU_1	*Faecalibacterium prausnitzii*	100
	OTU_479	*Faecalibacterium prausnitzii*	97
	OTU_122	*Ruminococcus callidus*	99
	OTU_4	*Gemmiger formicilis*	100
	OTU_48	*Ruminococcus bicirculans*	100
	OTU_68	*Clostridium leptum*	95
	OTU_87	*Clostridium methylpentosum*	90
	OTU_127	*Ruminococcus flavefaciens*	95
	OTU_22	*Ruminococcus bromii*	98
	OTU_3	*Faecalibacterium prausnitzii*	100
CR2	OTU_58	*Clostridium papyrosolvens*	87
	OTU_38	*Gemmiger formicilis*	94
	OTU_96	*Ruminococcus bromii*	90
	OTU_63	*Clostridium thermocellum*	89
	OTU_78	*Ruminococcus bromii*	86
	OTU_53	*Ruminococcus champanellensis*	90
	OTU_164	*Clostridium methylpentosum*	94
	OTU_67	*Clostridium leptum*	100
CR3	OTU_37	*Ruminococcus lactaris*	95

	OTU_33	*Clostridium thermocellum*	90
	OTU_93	*Clostridium papyrosolvens*	88
	OTU_15	*Clostridium viride*	90
	OTU_23	*Ruminococcus bicirculans*	87
	OTU_17	*Ruminococcus bromii*	88
	OTU_44	*Clostridium viride*	90
	OTU_461	*Clostridium viride*	92
	OTU_168	*Ruminococcus flavefaciens*	91
	OTU_81	*Clostridium thermocellum*	88
	OTU_103	*Ruminococcus albus*	90
	OTU_29	*Clostridium viride*	92
	OTU_155	*Ruminococcus albus*	91
	OTU_110	*Clostridium thermocellum*	90
	OTU_83	*Ruminococcus albus*	90
	OTU_8	*Ruminococcus bicirculans*	91
	OTU_108	*Faecalibacterium prausnitzii*	93
	OTU_86	*Ruminococcus champanellensis*	85
	OTU_10	*Clostridium viride*	91
	OTU_181	*Clostridium viride*	89
